# Individual pre-slaughter muscle proteolysis levels correlated with postmortem taste-related amino acid concentrations in broiler chickens

**DOI:** 10.1016/j.psj.2026.106553

**Published:** 2026-01-30

**Authors:** Sachi Katsumata, Minori Egawa, Koki Yoshino, Ayumi Katafuchi, Saki Shimamoto, Akira Ohtsuka, Daichi Ijiri

**Affiliations:** a Graduate School of Environmental, Life, Natural Science and Technology, Okayama University, Tsushima-naka, Okayama 700-8530, Japan; b Graduate School of Agriculture, Forestry and Fisheries, Kagoshima University, 1-21-24 Kagoshima 890-0065, Japan

**Keywords:** Pre-slaughter fasting, Skeletal muscle proteolysis, Free glutamic acid, Meat quality, Protease

## Abstract

Pre-slaughter fasting is an important practice in the poultry industry that reduces microbial contamination. However, fasting-induced skeletal muscle proteolysis can occur, which may influence meat quality because it increases the concentration of free amino acids that contribute to the umami taste during postmortem aging. In a previous study, we found that antemortem proteolysis levels induced by pre-slaughter fasting (0, 8, 16, and 24 h) were positively correlated with the free glutamic acid (Glu) content in the pectoralis major muscle after 48 h of aging. In this study, we investigated the relationship between individual differences in antemortem proteolysis levels and meat quality, especially free amino acids content and taste sensor value, in the pectoralis major muscle subjected to the same duration of 16-h pre-slaughter fasting, and the mechanisms of muscle free Glu accumulation during postmortem aging in broiler chickens. Antemortem skeletal muscle proteolysis levels, evaluated as changes in plasma N^τ^-methylhistidine concentrations, ranged from −1.0 to 12.7 nmol/mL (mean ± SD: 3.6 ± 3.01). Free Glu content in the pectoralis major muscle after 48 h of postmortem aging ranged from 9.7 to 45.8 mg/100 g (18.0 ± 7.34). A significant positive correlation was observed between antemortem proteolysis levels and postmortem free Glu content in the pectoralis major muscle after 48 h of aging (*r* = 0.42, *P* < 0.01). Postmortem free Glu content was positively correlated with the mRNA expression of ATP-independent proteolytic enzymes, including Calpain 11 (*r* = 0.27, *P* < 0.1), Calpain 2 (*r* = 0.45, *P* < 0.01), Cathepsin L-like (*r* = 0.55, *P* < 0.0001), and Cathepsin H (*r* = 0.35, *P* < 0.05). We measured the levels of troponin-T (TnT), which releases free Glu upon degradation, to investigate the cause of free Glu accumulation during aging. However, no correlation was observed between TnT and postmortem free Glu content. In contrast, specific low-molecular-weight proteins (approximately 12–15 kDa) exhibited associations with antemortem skeletal muscle proteolysis levels and postmortem free Glu acid content in the pectoralis major muscle. These findings suggest that individual pre-slaughter proteolysis levels influence postmortem muscle-free Glu accumulation by enhancing the expression of specific proteolytic enzymes or myofibrillar protein degradation.

## Highlights

• Antemortem muscle proteolysis levels differ among broilers despite identical fasting.
• Antemortem proteolysis level correlated with postmortem free glutamic acid.
• Free glutamic acid correlates with calpain and cathepsin mRNA expression.
• Low-molecular-weight proteins are linked to free glutamic acid accumulation.

## Introduction

Pre-slaughter fasting is a common practice in the poultry meat industry and reduces the risk of microbial contamination, ensuring the gastrointestinal contents of birds are removed (Lyon et al., 1991; Nijdam et al., 2005; Wang et al., 2013). However, fasting stress induces antemortem skeletal muscle proteolysis before slaughter by enhancing the activities of several muscle proteases and proteasomes (Buyse et al., 2002; Nakashima et al., 2005; Wang et al., 2013). In a previous study, we examined the effects of four pre-slaughter fasting durations (0, 8, 16, and 24 h) on antemortem skeletal muscle proteolysis and meat quality, and found that antemortem skeletal muscle proteolysis levels were positively correlated with free glutamic acid (Glu) content in the pectoralis major muscle after 48 h of postmortem aging, as well as with the umami taste and richness of chicken soup (Katsumata et al., 2024). These findings suggest that the skeletal muscle proteolysis levels in live animals before slaughter are strongly related to postmortem meat quality, especially free Glu and umami taste (Katsumata et al., 2024).

Antemortem skeletal muscle proteolysis is primarily regulated by the ubiquitin–proteasome and autophagy–lysosome pathways (Klionsky and Ohsumi, 1999; Voges et al., 1999). The ubiquitin–proteasome system (UPS) targets proteins for degradation by tagging them with polyubiquitin chains, leading to their breakdown by the 26S proteasome (Ciechanover, 1998; Hershko and Ciechanover, 1998). The autophagy-lysosomal pathway involves the key proteolytic enzymes calpains and cathepsins (Bechet et al., 2005). Postmortem muscle proteolysis plays a critical role in determining meat quality, including texture, flavor, and umami taste (Nishimura, 1998). ATP-dependent proteolytic systems, such as the 26S proteasome, are largely inactivated in postmortem skeletal muscles, whereas ATP-independent systems, including cathepsins and calpains, remain active and continue to contribute to muscle proteolysis (Nishimura, 1998). However, it remains unclear why antemortem proteolysis levels before slaughter are associated with postmortem free Glu accumulation during 48 h of aging. We hypothesized that some ATP-independent proteolytic enzymes are activated during the pre-slaughter fasting period, and consequently, the release of free Glu from muscle proteins is upregulated during the postmortem aging period.

Troponin T (TnT) is a myofibrillar protein that is highly susceptible to postmortem degradation and is broken down into a 30-kDa peptide during the aging of beef muscles (Macbride and Parrish, 1977; Olson and Parrish, 1977). The N-terminal regions of the bovine and porcine TnT isoforms are extremely rich in Glu residues (Kitamura et al., 2006). Postmortem proteolysis specifically cleaves this Glu-rich N-terminal region, leading to its release from the intact TnT protein (Muroya et al., 2004, 2007). Therefore, we focused on TnT and its degradation products as potential contributors to free Glu accumulation in the pectoralis major muscle after 48 h of aging.

This study aimed to investigate the relationship between individual differences in pre-slaughter skeletal muscle proteolysis levels and postmortem meat quality, particularly free amino acid content and taste in broiler chickens subjected to the same duration of pre-slaughter fasting. In addition, we examined the mechanisms responsible for increased Glu content, focusing on the involvement of proteasomes, proteases, and TnT degradation.

## Materials and methods

### Ethics statement

All experimental procedures were reviewed and approved by the Animal Care and Use Committee of Okayama University (approval number: OKU-2022965).

### Animals and experimental design

The experiment was conducted at the temperature- and humidity-controlled Animal Research Facility of Okayama University, Japan, during early summer (late April to June). Okayama is classified as a humid subtropical (Cfa) climate. A total of 50 0-day-old female broiler chicks (Chunky strain, Ross 308) were obtained from a commercial hatchery (Okayama, Japan). The animal experiment was repeated twice with 25 birds in consideration of animal welfare due to the cage size. They were housed in a wire-bottomed aluminum cage (2400 mm × 1000 mm) and provided with water and a semi-purified diet without animal protein. The diet was formulated according to the National Research Council (NRC) and fed according to the nutrient requirements for each growth stage (0–3 and 3–6 weeks of age) (Table 1). At 41 d of age, all chickens were weighed and fasted for 16 h. The room temperature was initially controlled at 30°C, and the temperature was decreased by 1°C every 2 days down to 25°C, and then it was maintained at 25°C. The lighting program used consisted of 20 h of light and 4 h of dark. Two chicks died during the experimental period; therefore, 48 chicks were used in this study. Statistical analyses revealed no significant differences in any of the measured parameters between the two replicates. As this study aimed to investigate individual differences, no control or treatment groups were established.

**Table 1:** Feed compositions used in this study.

	0-3 weeks of age	4-6 weeks of age
Ingredients (g/100 g)		
Corn grain	42.10	51.00
Soybean meal	44.70	37.30
Corn oil	8.80	7.30
DL-Methionine	0.19	0.25
CaHPO_4_	2.00	2.00
CaCO_3_	1.20	1.20
NaCl	0.50	0.50
Mineral and vitamin premix^1^	0.50	0.50
Nutrient contents		
Crude protein (%)	22.50	19.85
Metabolizable energy (Mcal/kg)	3.20	3.20

1 Content per kg of the vitamin and mineral premix: vitamin A = 90 mg, vitamin D3 = 1 mg, DL-alpha-tocopherol acetate = 2,000 mg, vitamin K3 = 229 mg, thiamin nitrate = 444 mg, riboflavin = 720 mg, calcium D-pantothenate = 2,174 mg, nicotinamide = 7,000 mg, pyridoxine hydrochloride = 700 mg, biotin = 30 mg, folic acid = 110 mg, cyanocobalamine = 2 mg, calcium iodinate = 108 mg, MgO = 198,991 mg, MnSO_4_ = 32,985 mg, ZnSO_4_ = 19,753 mg, FeSO_4_ = 43,523 mg, CuSO_4_ = 4019 mg, and choline chloride = 299,608 mg; Calculated following according to NRC.

### Sample collection

Blood samples were collected from the wing vein at 41 days of age before fasting and used to determine plasma N^τ^-methylhistidine concentrations. At 42 days of age, after body weight measurements, blood samples were collected from another wing vein to determine plasma N^τ^-methylhistidine concentrations after fasting. All the chickens were euthanized by decapitation under carbon dioxide anesthesia. Then, the chickens were dissected and the pectoralis major muscle, pectoralis minor muscle, liver, heart, and abdominal fat tissue depots were weighed. A portion of the left pectoralis major muscle was immediately frozen in liquid nitrogen and stored at −80°C until RNA and SDS-PAGE analysis. The right pectoralis major muscle was stored at 4°C for 48 h and used to determine drip loss, and a portion of the remaining half was stored at −80°C until the free amino acids, taste sensor traits, and SDS-PAGE analyses. All blood samples were collected in heparinized test tubes, centrifuged at 3,000 × *g* for 10 min at 4°C to separate the plasma, and stored at −80°C until analysis.

### Measurement of free amino acid levels

Free amino acid level analysis was performed as described previously (Katsumata et al., 2024). Briefly, aliquots of plasma samples (50 µL) and frozen pectoralis major muscles (100 mg) collected at slaughter and 48 h after aging were used for free amino acid analyses. Free N^τ^-methylhistidine and other free amino acids were separated and detected using the ultra-high-performance liquid chromatography (UHPLC) system (NexeraX2, Shimadzu Co., Ltd., Kyoto, Japan) with a Kinetex 2.6 µm column (EVO C18; 100 × 3.0 mm) and fluorescence detector (RF-20Axs), according to a previously described method for quantifying plasma-free amino acids (Azuma et al., 2016; Shiraishi et al., 2023; Tomita et al., 2022). Amino Acids Mixture Standard Solutions (Type H and Type B; 018-27881 and 011-27871, Fujifilm Wako, Osaka, Japan) were used. The antemortem change in individual plasma N^τ^-methylhistidine concentrations before and after fasting was calculated as the “concentration value prior to fasting ” minus the “concentration value immediately prior to slaughter,” and used as the skeletal muscle proteolysis level. The concentrations of free amino acids in the plasma were expressed in µmol/L, and those in the pectoralis major muscles were expressed as mg/100 g.

### mRNA extraction and quantitative real-time PCR

Pectoralis major muscle samples at slaughter were homogenized in ISOGEN II (317-07363, Nippon Gene, Tokyo, Japan) according to the manufacturer's instructions. Real-time polymerase chain reaction (PCR) was performed as previously described (Shimamoto et al., 2016a). Briefly, cDNA was synthesized from 50 ng of RNA per 1 μL of reaction solution using the PrimeScript RT Reagent Kit (RR036A, Takara, Shiga, Japan). Samples were incubated at 37°C for 15 min, 85°C for 5 s, and 4°C for 5 min. Gene expression was analyzed using real-time PCR with the 7300 Real-Time PCR system (Applied Biosystems, Foster City, CA, USA) with SYBR Select Master Mix (4472908, Applied Biosystems, Foster City, CA, USA). Thermal cycling conditions were as follows: initially maintained at 50°C for 2 min, 95°C for 2 min, and then 40 cycles at 95°C for 15 s, 55°C for 15 s, and 72°C for 1 min. 18S ribosomal RNA was used as an internal control. The following primers were used: Atrogin-1/ FBXO32, Murf-1/TRIM63, Calpain 11, Calpain 2, Cathepsin L-like, and Cathepsin H (Table 2).

**Table 2:** Primers used for qRT-PCR.

Gene	Sequences (5′−3′)	Accession No.
Atrogin-1/ FBXO32	Forward: CCAACAACCCAGAGACCTGT	NM_001389309.2
	Reverse: GGAGCTTCACACGAACATGA	
Murf-1/TRIM63	Forward: GGCAGGCTGGAGAAGATTGA	XM_015297754.3
	Reverse: ACCCTTTCGGGTTCTGTGTC	
Calpain 11	Forward: TACATCGTTGTGCCCTCCAC	NM_205303.1
	Reverse: TGCCGAGATCTCCTCATCCA	
Calpain 2	Forward: CTGGACACCGAGAAAACTTGGA	NM_205080.2
	Reverse: GTAAGCTTGTGCAAAGTTCCCA	
Cathepsin L-like	Forward: GGAATACCCTGGGATGGACG	NM_001281489.2
	Reverse: CCTCTCATTCGCCAGTCCAC	
Cathepsin H	Forward: GGGGCATGGATGGGTACTTC	NM_001318407.2
	Reverse: TCACACTAGAGGCACTGGGT	

### SDS-PAGE and western blotting analysis

SDS-PAGE analysis was performed as described previously, Huang et al. (2009) with slight modifications. Briefly, the pectoralis major muscle samples at slaughter and after 48 h of aging were homogenized in 7.5 volumes of PRB buffer (100 mM KCl, 2 mM MgCl_2_, 2 mM EGTA, 1 mM DTT, 1 mM NaN_3_, 2 mM Na_4_P_2_O_7_, 10 mM Tris-maleate, pH 6.8) with a blade-type homogenizer (Polytron PT 1200E, equipped with a high-foam PT-DA 12/2EC-E157 generator, Kinematica AG, Switzerland). The homogenate was centrifuged at 1,000 × *g* for 10 min and the pellet was washed twice with 10 volumes of low-salt buffer. In the last step, the myofibrils were suspended in Tris–EDTA buffer and immediately removed and mixed with treatment buffer (125 mM Tris, 4% SDS, and 20% glycerol); the samples were heated in a 50°C water bath for 20 min and then centrifuged for 30 min (19,000 × *g*). Protein concentration was determined using the Lowry method (Lowry et al., 1951), and the proteins were diluted to 4.0 μg/μL using treatment buffer, and then to 2.0 μg/μL using treatment buffer containing 10% MCE and 0.05% bromophenol blue. Samples were well mixed and heated at 50°C for 10 min and then stored at −80°C for subsequent SDS–PAGE.

The gels were run on an AE-6530 mini-slab electrophoresis unit (ATTO, Tokyo, Japan). The running buffer contained 25 mM Tris, 192 mM glycine, and 0.1% [w/v] SDS. The gels were loaded with 20 μg and run at a constant voltage of 200 V for approximately 1.2 h. To examine the protein bands, the gels were stained with Coomassie brilliant blue (11642, CBB stain One Super, Nacalai Tesque, Kyoto, Japan). All gels were analyzed using ImageJ software (National Institutes of Health, Bethesda, MD, USA), and the amount of protein was estimated. Antemortem abundance of protein bands was expressed as antemortem change in individual protein amounts before and after fasting, calculated as “amount value prior to the experiment” minus the “amount value immediately prior to slaughter.” Values were measured in µg/µL.

Western blotting was performed as described previously (Shimamoto et al., 2016b). The blocked membrane was incubated with a mouse monoclonal anti-troponin T (TnT) antibody (SAB4200717, Sigma-Aldrich, St. Louis, MO, USA).

### Taste sensor traits analysis

Taste traits analysis was performed as described previously (Katsumata et al., 2024). Briefly, chicken soup samples were prepared from the pectoralis major muscle after 48 h of aging according to the guidelines of Intelligent Sensor Technology. After defrosting and removing surface moisture from the pectoralis major muscles, 11 g of minced meat was weighed using a food mixer, and 110 mL of ultrapure water was added and incubated at 100°C for 60 min. Then, the broth was filtered through a filter paper (5A, Advantech, Tokyo, Japan) and used as the chicken soup sample.

### Statistical analysis

Pearson’s correlation coefficients were calculated to determine the relationship between antemortem skeletal muscle proteolysis levels, glutamic acid content, postmortem changes in protein band abundance in the pectoralis major muscles, and mRNA expression levels of proteases and taste sensor parameters. Analysis was performed using the PROC CORR procedure in SAS version 9.4 (SAS Institute, Cary, NC, USA). Differences were considered statistically significant at *P* < 0.05, and 0.05 ≤ *P* < 0.1 were interpreted as trends.

## Results

### Antemortem skeletal muscle proteolysis levels and glutamic acid content in pectoralis major muscles after aging

The average body and tissue weights are listed in Supplementary Table S1, and the drip loss and pH values are shown in Supplementary Table S2. All chickens showed a decrease in body weight during the 16 h fasting. The antemortem skeletal muscle proteolysis level (changes in plasma N^τ^-methylhistidine concentrations) was calculated as post-fasting minus pre-fasting, and it ranged from −1.0 to 12.7 nmol/mL (mean ± SD: 3.6 ± 3.01). Free Glu content in the pectoralis major muscles after 48 h of aging ranged from 9.7 to 45.8 mg/100 g (mean ± SD: 18.0 ± 7.34).

### Correlations between antemortem skeletal muscle proteolysis levels, free Glu content, and mRNA expression levels of muscle proteases

The free amino acid content of the pectoralis major muscle at slaughter and after 48 h of postmortem aging is shown in Table 3. There was no correlation between the free Glu content in the pectoralis major muscles at slaughter and antemortem skeletal muscle proteolysis levels. However, the change in free Glu content during postmortem aging, calculated as the difference between the values at 48 h postmortem and at slaughter, strongly correlated with antemortem skeletal muscle proteolysis levels (*r* = 0.55, *P* < 0.001) (Table 4). Consequently, the free Glu content in the pectoralis major muscles after 48 h of postmortem aging was strongly correlated with antemortem skeletal muscle proteolysis levels (*r* = 0.42, *P* < 0.01).

**Table 3:** Free amino acid contents in the pectoralis major muscle at slaughter and after 48 h of aging (mg/100 g; mean ± SE).

	At slaughter	After aging
Aspartic acid	2.35 ± 0.107	7.36 ± 0.415
Glutamic acid	8.48 ± 0.456	18.0 ± 1.07
Asparagine	2.29 ± 0.096	3.79 ± 0.140
Serine	10.2 ± 0.51	16.1 ± 1.00
Glutamine	13.6 ± 1.09	13.7 ± 0.94
Histidine	1.91 ± 0.179	1.61 ± 0.184
Glycine	26.0 ± 2.05	17.8 ± 1.28
Threonine	7.35 ± 0.534	5.69 ± 0.261
Arginine	16.7 ± 0.22	11.2 ± 0.54
Tyrosine	7.35 ± 0.226	6.25 ± 0.246
Valine	3.03 ± 0.166	6.16 ± 0.321
Methionine	1.43 ± 0.099	2.47 ± 0.153
Cystine	40.5 ± 2.27	61.2 ± 4.28
Tryptophane	1.92 ± 0.181	0.908 ± 0.0435
Phenylalanine	3.09 ± 0.127	6.07 ± 0.285
Isoleucine	1.91 ± 0.103	4.24 ± 0.234
Leucine	3.18 ± 0.161	8.64 ± 0.457
Lysine	6.45 ± 0.458	10.7 ± 0.78
Proline	6.52 ± 0.247	15.6 ± 1.57
N^τ^-methylhistidine	7.87 ± 0.722	9.17 ± 0.813
Beta-Alanine	6.13 ± 0.468	5.41 ± 0.419
Carnosine	262 ± 15.5	273 ± 13.7
Total free amino acids	159 ± 6.2	261 ± 8.9

**Table 4:** Pearson’s correlation between antemortem skeletal muscle proteolysis levels and Glu contents in the pectoralis major muscle after 48 h of postmortem aging in broiler chickens.

	Antemortem skeletal muscle proteolysis levels^1^	Postmortem Glu contents
Postmortem Glu contents	0.42⁎⁎	–
Antemortem skeletal muscle proteolysis levels^1^	–	0.42⁎⁎
Atrogin-1	0.39⁎⁎	–
Murf-1	0.34*	–
Calpain 11	0.42⁎⁎	0.27§
Calpain 2	–	0.45⁎⁎
Cathepsin L-like	–	0.55⁎⁎⁎
Cathepsin H	–	0.35 *

1 Antemortem skeletal muscle proteolysis levels were calculated as the change in individual plasma N^τ^-methylhistidine concentrations before and after fasting: “concentration value prior to the experiment” minus the “concentration value immediately prior to slaughter.”.

§ *P* < 0.1.

⁎ *P* < 0.05.

⁎⁎ *P* < 0.01.

⁎⁎⁎ *P* < 0.0001.

Furthermore, the mRNA expression levels of Atrogin-1 (*r* = 0.39, *P* < 0.01), Murf-1 (*r* = 0.34, *P* < 0.05), and Calpain 11 (*r* = 0.42, *P* < 0.001) were positively correlated with antemortem skeletal muscle proteolysis levels (Table 4). Additionally, the free Glu content in the pectoralis major muscles after 48 h of aging was positively correlated with the mRNA expression levels of Calpain 11 (*r* = 0.27, *P* = 0.06), Calpain 2 (*r* = 0.45, *P* < 0.05), Cathepsin L-like (*r* = 0.55, *P* < 0.0001), and Cathepsin H (*r* = 0.35, *P* < 0.05) (Table 4).

### Postmortem changes in the proteolytic pattern of myofibrillar protein

**Fig. 1:**
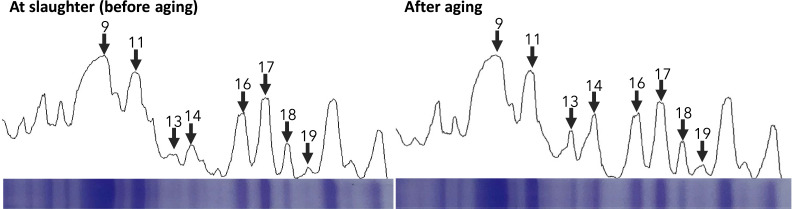
Densitometric profiles of SDS-PAGE at slaughter and after aging.

**Fig. 2:**
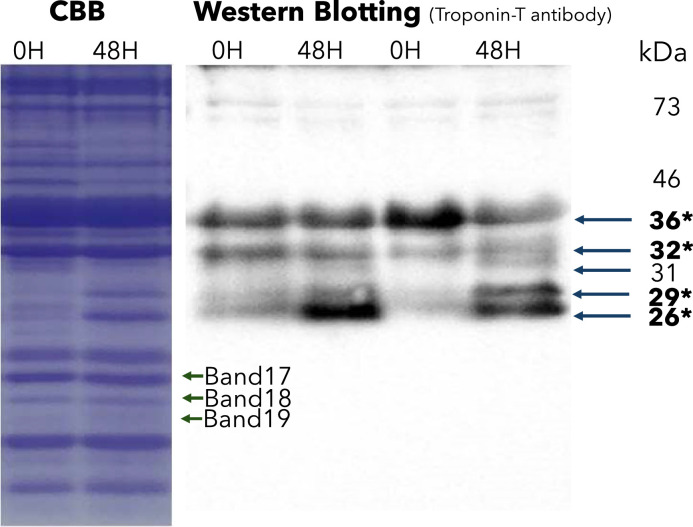
Results of SDS-PAGE and Western Blotting.

SDS-PAGE and western blot analyses were used to investigate the relationship between TnT, its degradation products, and free Glu content in the pectoralis major muscle after 48 h of aging. Band intensities were measured by densitometry, and the area of each protein band was quantified from the chromatogram using the ImageJ software (Fig. 1). As a result, in the pectoralis major muscle after 48 h of postmortem aging, the area of the five protein bands increased, while that of the 17 bands decreased compared to those after fasting. Immunoblot assays with rabbit anti-TnT monoclonal antibody identified band 9 as intact TnT and bands 11, 13, and 14 as degradation products (Fig. 2).

All gels were stained at Coomassie brilliant blue and analyzed with ImageJ software. Band 9: Actin; Band 11: Troponin-T; Band 13 and 14: Troponin T degradation protein; Band 17: unknown (15 kDa); Band 18: unknown (13 kDa); Band 19: unknown (12 kDa)

*36 kDa: Troponin-T (Band 9) 32 kDa: Troponin-T degradation protein (Band 11) 29 kDa: Troponin-T degradation protein (Band 13) 26 kDa: Troponin-T degradation protein (Band14)

### Correlation analysis of postmortem change in myofibrillar protein, and glutamic acid contents, proteases mRNA expression, and taste sensor traits

The antemortem skeletal muscle proteolysis levels and free Glu content in the pectoralis major muscles after 48 h of postmortem aging were not correlated with postmortem changes in band 9 (TnT) or bands 11, 13, and 14 (degradation products of TnT). However, postmortem change in the band 19 showed positive correlations with the antemortem skeletal muscle proteolysis levels (*r* = 0.32, *P* < 0.05), free Glu content (*r* = 0.56, *P* < 0.001) and total free amino acids content (*r* = 0.40, *P* < 0.01), mRNA expression levels of Calpain 11 (*r* = 0.31, *P* < 0.01), Calpain 2 (*r* = 0.38, *P* < 0.01), Cathepsin L-like (*r* = 0.34, *P* < 0.05), and Cathepsin H (*r* = 0.46, *P* < 0.01) as well as umami taste (*r* = 0.40, *P* < 0.01) and bitterness and off-taste (*r* = 0.38, *P* < 0.05) (Table 5). In contrast, postmortem changes in band 17 were negatively correlated with the antemortem skeletal muscle proteolysis levels (*r* = −0.38, *P* < 0.01), free Glu content (*r* = −0.53, *P* < 0.001) and total free amino acids content (*r* = −0.41, *P* < 0.01), umami taste (*r* = −0.46, *P* < 0.01), bitterness and off-taste (*r* = −0.40, *P* < 0.01), as well as mRNA expression levels of Calpain 2 (*r* = −0.52, *P* < 0.001), Cathepsin L-like (*r* = −0.42, *P* < 0.0001), and Cathepsin H (*r* = −0.45, *P* < 0.01) in the pectoralis major muscles at slaughter (Table 5). Similarly, the postmortem change in band 18 was negatively correlated with the antemortem skeletal muscle proteolysis levels (*r* = −0.30, *P* < 0.05), free Glu content (*r* = −0.38, *P* < 0.01), total free amino acid content (*r* = −0.33, *P* < 0.05), and mRNA expression levels of Calpain 2 (*r* = −0.31, *P* < 0.05), Cathepsin L-like (*r* = −0.39, *P* < 0.001), and Cathepsin H (*r* = −0.31, *P* < 0.05) (Table 5). Postmortem changes in band 16 were negatively correlated with umami taste (*r* = −0.30, *P* < 0.05), and bitterness and off-taste (*r* = −0.36, *P* < 0.05) (Table 5).

**Table 5:** Pearson’s correlation between antemortem abundance of protein bands, antemortem skeletal muscle proteolysis levels, and Glu contents in the pectoralis major muscle after 48 h of postmortem aging in broiler chickens.

	Band 16^1^	Band 17^1^	Band 18^1^	Band 19^1^
Antemortem skeletal muscle proteolysis level 2	–	−0.38⁎⁎	−0.30*	0.32*
Postmortem pectoralis major muscle				
Umami taste	−0.30*	−0.46⁎⁎	–	0.40⁎⁎
Bitterness and off-taste	−0.36*	−0.40⁎⁎	–	0.38⁎⁎
Free Glu contents	–	−0.53⁎⁎⁎	−0.38⁎⁎	0.56⁎⁎⁎
Total free amino acids contents	–	−0.41⁎⁎	−0.33*	0.40⁎⁎
mRNA expression levels				
Calpain 11	–	–	–	0.31*
Calpain 2	–	−0.52⁎⁎	−0.31*	0.38⁎⁎
Cathepsin L-like	–	−0.42⁎⁎	−0.39⁎⁎	0.34*
Cathepsin H	–	−0.45*	−0.31*	0.46⁎⁎

1 Antemortem abundance of protein bands was expressed as the change in individual protein amounts before and after fasting: “amount value prior to the experiment” minus the “amount value immediately prior to slaughter.” Values are presented in µg/µL.

2 Antemortem skeletal muscle proteolysis levels were calculated as the change in individual plasma N^τ^-methylhistidine concentrations before and after fasting: “concentration value prior to the experiment” minus the “concentration value immediately prior to slaughter.”.

⁎ *P* < 0.05.

⁎⁎ *P* < 0.01.

⁎⁎⁎ *P* < 0.0001.

## Discussion

Pre-slaughter fasting is a crucial practice in the poultry meat industry from a food hygiene perspective. In our previous study, we found that the antemortem skeletal muscle proteolysis level induced by pre-slaughter fasting (0, 8, 16, and 24 h) was positively correlated with free Glu content in the pectoralis major muscles after 48 h of postmortem aging (Katsumata et al., 2024). In the present study, despite all broilers undergoing the same 16-h pre-slaughter fasting, we observed a significant correlation between individual antemortem skeletal muscle proteolysis levels and changes in their muscle free Glu content and final muscle free Glu content after 48 h of postmortem aging (Table 4); however, no correlation was observed between the antemortem skeletal muscle proteolysis levels and free Glu content in the pectoralis major muscles at slaughter. These results suggest that individual differences in the free Glu content in meat are strongly associated with antemortem skeletal muscle proteolysis levels.

We conducted further analyses to elucidate the molecular mechanisms linking antemortem muscle proteolysis and free Glu accumulation during 48 h of postmortem aging. Antemortem skeletal muscle proteolysis is primarily regulated by the ATP-dependent UPS, whereas the ATP-independent autophagy–lysosome pathway plays a major role in postmortem skeletal muscle degradation (Bechet et al., 2005; Klionsky and Ohsumi, 1999; Lord et al., 2024; Tesseraud et al., 2021; Voges et al., 1999). In this study, we confirmed that antemortem skeletal muscle proteolysis levels were positively correlated with the expression of Atrogin-1, Murf-1, and Calpain 11 (Table 4). Additionally, the free Glu content in the pectoralis major muscle after 48 h of postmortem aging was positively correlated with Calpain 11 and 2, as well as Cathepsin L-like and H (Table 4). Calpain 11 was positively correlated with the free Glu content after 48 h of postmortem aging, whereas Atrogin-1 and Murf-1 did not show any correlation. These results suggest that the UPS primarily contributes to fasting-induced increases in antemortem skeletal muscle proteolysis, while Calpain 11 retains its activity throughout the antemortem and postmortem periods, contributing to free Glu accumulation during aging. Calpain 11 is specific to the testis in eutherians; however, in avian species, Calpain 11 ubiquitously forms the conventional μ/m-Calpain and thus has the same function as μ/m-Calpain (Ono and Sorimachi, 2012; Ono et al., 2016; Sorimachi et al., 1995). This suggests that Calpain 11 may also play a comparable role in postmortem muscle proteolysis (Ono and Sorimachi, 2012; Ono et al., 2016; Sorimachi et al., 1995). A previous study reported that μ- and m-Calpain mRNA expression in chick skeletal muscles increased following fasting (Nakashima et al., 2005). In this study, Calpain 11 and other ATP-independent autophagy–lysosome pathway factors, including Calpain 2, Cathepsin L-like, and Cathepsin H, may play key roles in free Glu accumulation during aging; in particular, Calpain 11 may function in the transition from antemortem to postmortem.

We next investigated which specific muscle fiber proteins are targeted by ATP-independent enzymes, such as Calpain 11 and 2, as well as Cathepsin L-like, and H, as their degradation can contribute to the release and accumulation of free Glu during postmortem aging. We focused on TnT and its degradation product because TnT is known to be degraded into a 30-kDa peptide during postmortem aging, particularly through cleavage of its Glu-rich N-terminal region (Kitamura et al., 2006; Macbride and Parrish, 1977; Muroya et al., 2004, 2007; Olson and Parrish, 1977; Rasmussen and Jin, 2021). This region may contribute to free Glu accumulation in muscles during postmortem aging. Therefore, SDS-PAGE and western blotting analyses were performed to evaluate TnT and its degradation products. No significant correlation was found between the free Glu content after 48 h of aging and TnT (band 13) abundance in the SDS-PAGE analysis (Fig. 2; Table 5).

Interestingly, band 19, which appeared at a lower molecular weight than TnT but was not recognized by the TnT monoclonal antibody, showed significant positive correlations with antemortem skeletal muscle proteolysis levels, free Glu content after 48 h of postmortem aging, umami taste, and the mRNA levels of Calpain 11, Calpain 2, Cathepsin L-like, and Cathepsin H (Table 5). In contrast, bands 17 and 18 were negatively correlated with these parameters (Table 5). These results suggest that the degradation of bands 17 and 18 may contribute to free Glu accumulation in the pectoralis major muscle during aging, whereas band 19 may represent a degradation product associated with increased free Glu. Although the identities of these bands remain unclear, they appear to contribute significantly to the accumulation of free Glu in the muscles during postmortem aging.

Further studies are needed to identify bands 17, 18, and 19, as well as other proteins in the pectoralis major muscle that yield 12-15 kDa protein fragments enriched in Glu during postmortem aging. To our knowledge, this is the first study to demonstrate that individual differences in antemortem muscle proteolysis levels, induced by pre-slaughter fasting, are associated with specific low-molecular-weight proteins that may contribute to postmortem free Glu accumulation and umami taste in the pectoralis major muscle after 48 h of aging.

## CRediT authorship contribution statement

**Sachi Katsumata:** Writing – original draft, Visualization, Investigation, Formal analysis, Data curation, Conceptualization. **Minori Egawa:** Writing – review & editing, Visualization, Investigation, Data curation. **Koki Yoshino:** Writing – review & editing, Visualization, Investigation, Data curation. **Ayumi Katafuchi:** Writing – review & editing, Investigation. **Saki Shimamoto:** Writing – review & editing, Resources. **Akira Ohtsuka:** Writing – review & editing, Resources. **Daichi Ijiri:** Writing – review & editing, Visualization, Supervision, Resources, Investigation, Data curation, Conceptualization.

## Disclosures

All authors certify that they have no affiliations with or involvement in any organization or entity with any financial interest or non-financial interest in the subject matter or materials discussed in this manuscript.

## Appendix Supplementary materials

Image, application 1
