# Supplementary material for: Individual pre-slaughter muscle proteolysis levels correlated with postmortem taste-related amino acid concentrations in broiler chickens
Source: Poult Sci. 2026 Jan 30;105(5):106553. doi: 10.1016/j.psj.2026.106553 (PMC12917532; doi:10.1016/j.psj.2026.106553)
Supplement: Supplementary file 1 [file mmc1.docx]

**Individual pre-slaughter muscle proteolysis levels correlated with postmortem taste-related amino acid concentrations in broiler chickens**

Sachi Katsumata*, Minori Egawa*, Koki Yoshino*, Ayumi Katafuchi^#^, Saki Shimamoto^#,^ Akira Ohtsuka^#^, Daichi Ijiri^#^

* Graduate School of Environmental, Life, Natural Science and Technology, Okayama University, Tsushima-naka, Okayama 700-8530, Japan

^#^ Graduate School of Agriculture, Forestry and Fisheries, Kagoshima University, 1-21-24 Kagoshima 890-0065, Japan

---------------------------------

Supplementary Table S1. Body weights and weights of tissue (g).

|  | Average (g) | Standard error |
| --- | --- | --- |
| Body weight at slaughter | 2116.3 | 33.3 |
| Body weight change^＊^ | -80.7 | 3.3 |
| Pectoralis major muscle | 402.0 | 10.3 |
| Pectoralis minor muscle | 91.6 | 2.0 |
| Tight muscle | 425.9 | 7.8 |
| Liver | 36.6 | 0.9 |
| Heart | 7.1 | 0.2 |
| Abdominal fat tissue | 23.1 | 1.7 |

* Body weights change; body weight at slaughter – body weight just before fasting treatment (g).

Supplementary Table S2. pH and drip loss in experimental chicken meat.

|  | Average ± Standard error | |
| --- | --- | --- |
|  | At slaughter | At 48h of aging |
| pH | 6.5 8 ± 0.28 | 5.81 ± 0.13 |
|  |  |  |
| Drip loss (%)^§^ | 1.14 ± 0.71 | |

^§^ Drip loss = (Pectoralis major muscle weight at slaughter − Pectoralis major muscle weight after 48 hours of aging) / Pectoralis major muscle weight at slaughter×100
